# PTH1R translocation to primary cilia in mechanically‐stimulated ostecytes prevents osteoclast formation via regulation of CXCL5 and IL‐6 secretion

**DOI:** 10.1002/jcp.30849

**Published:** 2022-08-07

**Authors:** Irene Tirado‐Cabrera, Eduardo Martin‐Guerrero, Sara Heredero‐Jimenez, Juan A. Ardura, Arancha R. Gortázar

**Affiliations:** ^1^ Bone Physiopathology Laboratory, Applied Molecular Medicine Institute (IMMA), Universidad San Pablo‐CEU, CEU Universities Campus Monteprincipe Alcorcón Spain; ^2^ Department of Basic Medical Sciences, School of Medicine, Universidad San Pablo‐CEU, CEU Universities Campus Monteprincipe Alcorcón Madrid Spain

**Keywords:** mechanotransduction, osteoclasts, osteocytes, primary cilia, PTH1R

## Abstract

Osteocytes respond to mechanical forces controlling osteoblast and osteoclast function. Mechanical stimulation decreases osteocyte apoptosis and promotes bone formation. Primary cilia have been described as potential mechanosensors in bone cells. Certain osteogenic responses induced by fluid flow (FF) in vitro are decreased by primary cilia inhibition in MLO‐Y4 osteocytes. The parathyroid hormone (PTH) receptor type 1 (PTH1R) modulates osteoblast, osteoclast, and osteocyte effects upon activation by PTH or PTH‐related protein (PTHrP) in osteoblastic cells. Moreover, some actions of PTH1R seem to be triggered directly by mechanical stimulation. We hypothesize that PTH1R forms a signaling complex in the primary cilium that is essential for mechanotransduction in osteocytes and affects osteocyte‐osteoclast communication. MLO‐Y4 osteocytes were stimulated by FF or PTHrP (1−37). PTH1R and primary cilia signaling were abrogated using PTH1R or primary cilia specific siRNAs or inhibitors, respectively. Conditioned media obtained from mechanically‐ or PTHrP‐stimulated MLO‐Y4 cells inhibited the migration of preosteoclastic cells and osteoclast differentiation. Redistribution of PTH1R along the entire cilium was observed in mechanically stimulated MLO‐Y4 osteocytic cells. Preincubation of MLO‐Y4 cells with the Gli‐1 antagonist, the adenylate cyclase inhibitor (SQ22536), or with the phospholipase C inhibitor (U73122), affected the migration of osteoclast precursors and osteoclastogenesis. Proteomic analysis and neutralizing experiments showed that FF and PTH1R activation control osteoclast function through the modulation of C‐X‐C Motif Chemokine Ligand 5 (CXCL5) and interleukin‐6 (IL‐6) secretion in osteocytes. These novel findings indicate that both primary cilium and PTH1R are necessary in osteocytes for proper communication with osteoclasts and show that mechanical stimulation inhibits osteoclast recruitment and differentiation through CXCL5, while PTH1R activation regulate these processes via IL‐6.

## 1 INTRODUCTION

Mechanical loading is a key regulator of bone formation and maintenance. Osteocytes, the most abundant cells in bone, respond to mechanical stimulation altering the release of molecules that regulate osteoclast and osteoblast function (Delgado‐Calle, Sato et al., 2017; Wijenayaka et al., 2011; You et al., 2008). In the absence of mechanical stimulation, osteocytes produce signals which induce the recruitment and differentiation of osteoclast precursors stimulating bone resorption (Plotkin et al., 2015).

PTH1R, a family B G‐protein‐coupled receptor (GPCR) that can potentially trigger several intracellular signaling pathways in bone, plays a pivotal role in bone formation and remodeling (Powell WF et al., 2011; Rhee et al., 2011). Recently, we and others have described that mechanical stimulation can directly activate the PTH1R without ligand stimulation, indicating the potential role of the parathyroid hormone (PTH) receptor as a mechanosensor in osteocytes and osteoblasts (Maycas et al., 2015a; Zhang & Frangos, 2009). In this regard, our group has demonstrated that PTH1R is an important component of the mechanical signal transduction machinery in MLO‐Y4 osteocytic cells. In these cells, ligand‐independent PTH1R activation occurred immediately after mechanical stimulation by fluid flow (FF) (Maycas et al., 2015a). Moreover, FF induced an increase of PTH1R presence at the plasma membrane of MLO‐Y4 cells, and, in vivo, mechanical loading, in combination with short treatment with PTHrP‐derived peptides, prevented bone fragility induced by diabetes (Maycas et al., 2015, 2017).

Primary cilia are nonmotile microtubule‐based organelles that extend from the apical surface of nearly every cell in the human body, such as kidney epithelial cells or bone cells (J. Malicki & Johnson, 2017). Primary cilia behave as a cellular “antenna” that receive diverse extracellular signals including mechanical stimuli and chemical signals and translate them into intracellular signaling responses (Ding et al., 2020; J. J. Malicki & Johnson, 2017; P. Wang et al., 2019). The spatiotemporal localization of specific receptors and associated signaling components along the cilio‐centrosome axis, including proteins involved in the regulation of bone remodeling such as hedgehog and several receptors coupled to G proteins (GPCRs) class A, B, and F, make possible its sensing abilities (Schou et al., 2015). It has been shown that primary cilia of osteocyte‐like MLO‐Y4 cells and osteoblast‐like MC3T3‐E1 cells extend from the cell surface and deflect during FF. Nevertheless, the cellular responses of osteoblasts and osteocytes to FF was significantly inhibited after the abrogation of primary cilium formation and function (Hoey et al., 2011; Malone et al., 2007). Primary cilia in osteocytes are responsible for detecting and mediating electromagnetic stimulation signals and thus regulate osteoclastic activity and function (P. Wang et al., 2019). The recent description of the role of PTH1R as a mechanosensor at the cell membrane of osteocytes and osteoblasts (Maycas et al., 2015a; Tu et al., 2015), suggests that this GPCR receptor could be especially abundant in an organelle focused to the detection of mechanical stimuli such as the primary cilium (Martín‐Guerrero et al., 2020). In fact, PTH1R induces prosurvival actions via primary cilia‐ and Gli‐1‐dependent mechanism in both osteocytes and osteoblasts (Martín‐Guerrero et al., 2020). However, whether PTH1R can be mobilized to the primary cilia during mechanical stimulation and how this translocation affects osteocyte‐osteoclast communication is not known.

In the present study, we show that mechanical stimulation of osteocytes prevents osteoclast activation after PTH1R translocation to primary cilium through the inhibition of C‐X‐C Motif Chemokine Ligand 5 (CXCL5) secretion. Moreover, lack of PTH1R expression induces osteoclast migration and formation by an interleukin‐6 (IL‐6)‐dependent pathway. These findings suggest a mechanism, based on PTH1R mobilization to the primary cilia, that could explain the therapeutic efficiency of combined PTH or PTHrP treatment and mechanical stimulation to preserve bone mass and quality.

## 2 MATERIALS AND METHODS

### 2.1 Cell culture

Mouse osteocytic MLO‐Y4 cells (generously donated by Dr. Lynda Bonewald) were grown in α‐MEM supplemented with 2.5% fetal calf serum (FCS) and 2.5% fetal bovine serum (FBS). Murine monocyte cell line (RAW 264.7) was purchased from the American Type Culture Collection and cultured in Dulbecco's Modified Eagle's Medium (DMEM) supplemented with 10% FBS. All cells were cultured with penicillin (100 units/ml) and streptomycin (100 μg/ml) in a 5% CO_2_ humidified incubator at 37°C. MLO‐Y4 cells were plated at 5 × 10^5^ cells/cm^2^ on collagen‐coated glass slides (FlexCell); the next day, fresh medium with 1% FBS was added for 24 h. Then, cells were submitted or not (static control, SC) to mechanical stress by laminar FF with a shear stress of 10 dynes/cm^2^ for 10 min in a Flexcell® Streamer® shear stress device. The media flowed over the cells was consistently serum‐depleted α‐MEM in every experimental condition. Or they were stimulated by PTHrP (1‐37) (Bachem) for 10 min. Cells were cultured with fresh medium (α‐MEM without serum) for an additional 18 h after being stimulates with PTHrP (1‐37), FF or SC to collect the cell conditioned medium (CM) in the different experimental groups. RNA was isolated after 6 h of the stimulation with PTHrP (1‐37),  FF, or SC.

### 2.2 Inhibition of primary cilia, PTH1R, Gl1, cyclic AMP, and phospholipase C

Primary cilia and PTH1R were inhibited using two different approaches. Cells were treated with 1 mM aqueous chloral hydrate or with 100 nM PTHrP (7‐34)  for 1 h, respectively. The other approach was transfected with a mixture of three small interfering RNAs (siRNAs; each at 20 nM) against different coding target sequences of mouseIFT88 (186729; 186730; 186731; Thermo Fisher Scientific) and against different coding target sequences of mousePTH1R (151326; 68886; 68980; Thermo Fisher Scientific) for 24 h, respectively. Transfection of siRNAs was done using lipofectamine RNAiMax (Life Technologies) overnight at 37°C, according to the manufacturer's instructions. A scrambled sequence (control siRNA‐A; Santa Cruz Biotechnology) was used as a negative control for evaluating RNA interference off‐targeted effects and to verify the accuracy of gene‐specific siRNA‐dependent changes in the different parameters evaluated. Gli transcription factor was inhibited using 10 μM Gli‐1‐Antagonist 61 (GANT61; Santa Cruz Biotechnology) for 1 h when appropriate. cAMP and phospholipase C were inhibited with 100 μM of the adenylate cyclase inhibitor SQ22536 or with 1 μM of the phospholipase C inhibitor U73122, respectively.

### 2.3 Cell transfection

MLO‐Y4 cells were transfected with 1 μg of a human green fluorescent protein (GFP)‐PTH1R construct (generously donated by Dr. Peter Friedman) using lipofectamine 3000 (Life Technologies) for 4 h at 37°C, according to the manufacturer's guidelines.

### 2.4 Inmunofluorescence

Cells were fixed with 4% p‐formaldehyde (for α‐acetylated tubulin) and permeabilized using 0.5% Triton in phosphate‐buffered saline (PBS), pH 7.4. Nonspecific binding was blocked with 5% goat serum in bovine serum albumin, followed by overnight incubation at 4°C with mouse α‐acetylated tubulin antibody (Sigma Aldrich) at dilution 1/1000. Cells were rinsed three times with PBS before incubation for 1 h with Alexa fluor 546‐conjugated anti‐mouse immunoglobulin G (IgG) secondary antibodies (Invitrogen) at dilution 1/1000. Samples were mounted in FluorSafe Reagent (Calbiochem) and examined using a Leica DMI 8 confocal microscope. Colocalization of PTH1R with primary cilia was counted from cells transfected with a GFP‐PTH1R plasmid in each condition. It was also taken into account if this colocalization was in the whole cilium or only occurred in the base of the primary cilium.

### 2.5 Proteomic analysis

CM obtained from osteocytes +/‐ mechanical stimulation +/‐ PTH1R inhibition was analyzed by tandem mass tag (TMT) proteomics. Proteins secreted by osteocytes under these different conditions were relatively quantified and compared by the protein chemistry facility of CBMSO (Centro de Biología Molecular Severo Ochoa). In‐Gel Digestion (Stacking gel) was performed as previously described Moreno M. Moreno et al. (2014). TMT labeling and high pH fractionation were carried out as following. The peptide mixture from desalted proteins tryptic digest (50 µg) was labeled using chemicals from the TMT reagent sixplex Isobaric Mass Tagging Kit (reagents 126, 127, 128, and 129) (Thermo Fisher Scientific) essentially as described by manufacturer's. Peptides were dissolved in 50 μl of 100 mM triethylammonium bicarbonate, adjusted to pH 8. For labeling, each TMT reagent was dissolved in 41 μl of acetonitrile and added to the respective peptide mixture and then incubated at room temperature for 1 h. Labeling was stopped by the addition of 8 μl 5% hidroxilamine. Whole supernatants were dried down and the samples mixed to obtain the “4plex‐labeled mixture.” The mixture was analyzed by RP‐LC‐MS/MS to check the efficiency of the labeling. The sample was then fractionated using the high‐pH, reversed‐phase peptide fractionation kit (Pierce) as described with minor modifications. The sample reswollen in 0.1% TFA and then, loaded onto an equilibrated, high‐pH, reversed‐phase fractionation spin column. A step gradient of increasing acetonitrile concentrations in a volatile high pH solution was then applied to the columns to elute bound peptides into nine different fractions (5%−80% acetonitrile) collected by centrifugation. The fractions obtained from high‐pH, reversed‐phase 4plex‐labeled mixture were dried and stored until analysis by mass spectrometry for quantification. Quantitative analysis was performed by reverse phase‐liquid chromatography RP‐LC‐MS/MS as previously described Alonso Alonso et al. (2015). Peptide identification from raw data (a single search was performed with all nine raws from the fractionation) was carried out using PEAKS Studio X search engine (Bioinformatics Solutions Inc.). Database search was performed against uniprot‐Mus musculus.fasta (decoy‐fusion database).

### 2.6 Enzyme‐linked immunosorbent assay (ELISA)

IL‐6 and CXCL5 were evaluated in MLO‐Y4 cell‐CM using specific mouse ELISA Kits from Thermo Fisher Scientific, according to the corresponding manufacturer's instructions. Briefly, CM were collected and centrifuged at 1000 rpm for 10 min to pellet cellular debris. The optical absorbance of each well was measured in an ELISA reader (Varioskan Lux; Thermo Fisher Scientific) at 450 nm. The sensitivity of the assays was 6.5 pg/ml for IL‐6 and 35 pg/ml for CXCL5, and their inter assay variation coefficient was <12%.

### 2.7 Isolation of peripheral blood mononuclear cells (PBMCs) from buffy coats

PBMCs for generating human osteoclasts in vitro were isolated from buffy coats. Buffy coat was diluted with an equal volume of RPMI, carefully layer 30 ml of the diluted buffy coat on to 15 ml Ficoll and centrifuge at 800*g* for 40 min, with brake set to zero. Mononuclear cell layer from each tube was collected and transferred to two new 50 ml tubes. PBS was added to a volume of 45 ml per tube to wash the cells and centrifuge at 250*g* for 10 min. Cells were grown in α‐MEM supplemented with 10% FCS with penicillin (100 units/ml) and streptomycin (100 μg/ml) in a 5% CO_2_ humidified incubator at 37°C.

### 2.8 Transwell migration assay

Migration assay was performed using Costar transwell cell culture chamber inserts (Corning Costar Corporation) with an 8 µm pore size. Briefly, 5 × 10^4^ RAW 264.7 cells or human osteoclasts from buffy coat were placed in the upper chamber in 200 µl of DMEM supplemented with 10% FBS or α‐MEM supplemented with 10% FCS, respectively. Standard medium with 1% FBS or FCS and containing 20% of each MLO‐Y4 cell‐conditioned medium for 18 h were placed in the lower compartment. Furthermore, in others experiments, 2 µg/ml of neutralizing antibody anti‐mCXCL5 (Thermo Fisher Scientific) and 1 µg/ml of anti‐mIL‐6 (R&D Systems) were used. Then, the medium and the upper cell layer were removed with the aid of a cotton swab, and cells on the underside of the transwell were fixed with PFA 4%, stained with crystal violet (Sigma Aldrich) and counted in five randomly selected fields at X200 magnification using Leica DM 5500B microscope.

### 2.9 Generation of osteoclasts for assessment of osteoclastogenesis

Cells from buffy coat were grown in α‐MEM supplemented with 10% FCS containing 20 ng/ml of macrophage colony‐stimulating factor (M‐CSF), feeding with fresh media until they reach a suitable confluency. Briefly, 2 × 10^4^ human osteoclasts from buffy coat were seeded into wells of a 96‐well plate, supplemented with α‐MEM containing 20 ng/ml M‐CSF plus 20 ng/ml of receptor activator of nuclear factor kB ligand (RANKL). As a control, cells were incubated without RANKL were included. The following day, cells were fed with fresh α‐MEM containing M‐CSF and RANKL as appropriate, plus the accurate treatments; 20% of each MLO‐Y4 cell‐conditioned medium. Moreover, in some experiments, neutralizing antibody anti‐mCXCL5 (Thermo Fisher Scientific) and anti‐mIL‐6 (R&D Systems) were used. After 3 days, the medium was removed, cells were rinsed off with PBS to eliminate nonadherent cells. Cells were fixed with 4% p‐formaldehyde, permeabilized with 100% methanol for 20 min and stained with hematoxylin for 5 min. The differentiation of human monocytic cells into osteoclasts were determined by morphology, observing the formation of giant cells with at least three or more nuclei.

### 2.10 Statistical analysis

Data are expressed as means ± standard deviation. Statistical analysis was performed using GraphPad Prism (GraphPad software). Differences among conditions were evaluated by nonparametric variance analysis (Kruskal−Wallis) followed by Mann−Whitney test. The *p* < 0.05 was considered significant.

## 3 RESULTS

### 3.1 Secretome of mechanically‐stimulated osteocytes inhibits the migration of preosteoclastic cells and osteoclast differentiation by a mechanism dependent on the primary cilium and PTH1R

**Figure 1:**
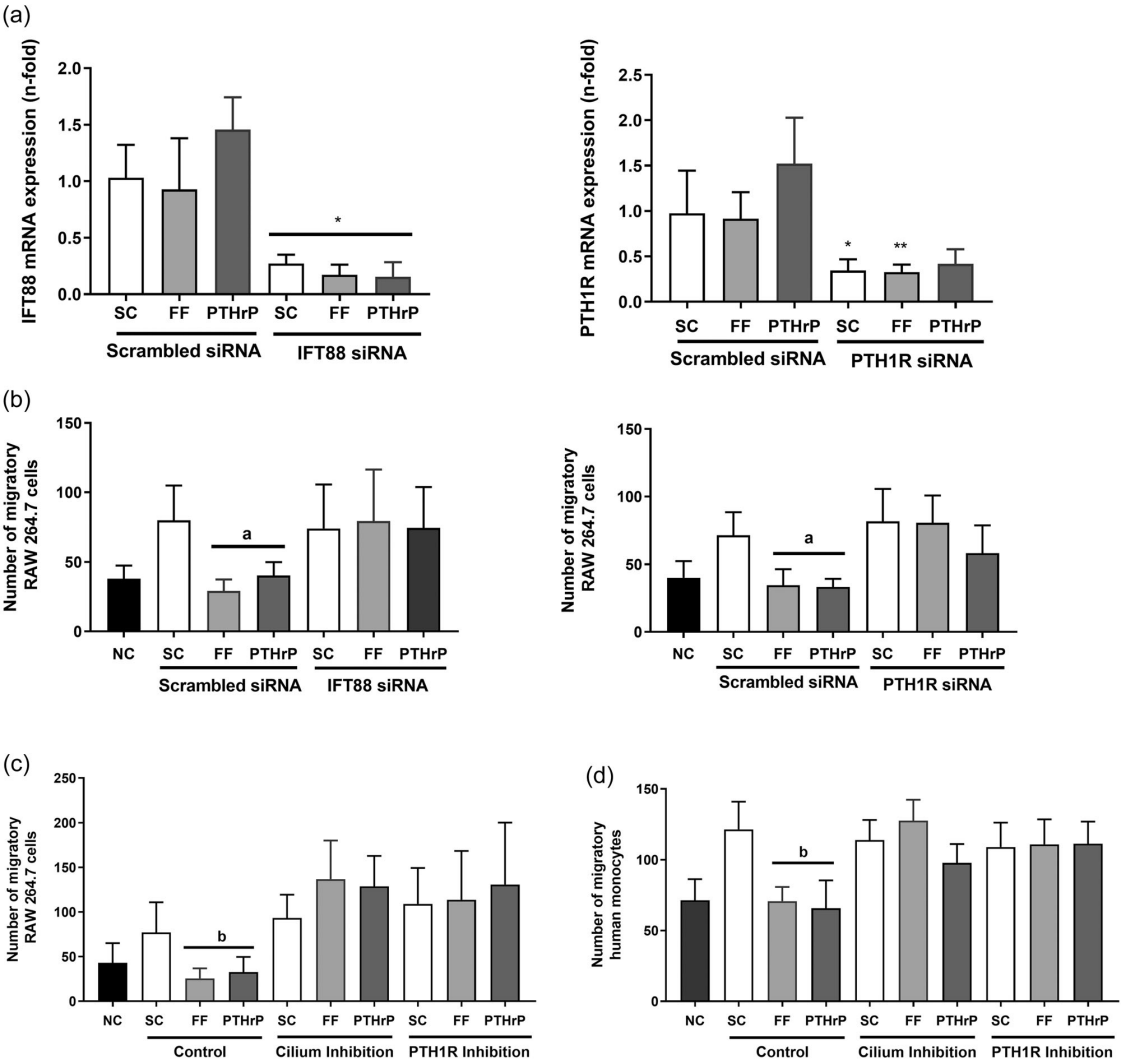
CM from mechanically‐stimulated MLO‐Y4 cells inhibit the migration of monocytes by a mechanism dependent on the primary cilium and PTH1R. MLO‐Y4 osteocytic cells were transfected with three IFT88, three PTH1R siRNAs, or with a scrambled siRNA for 24 h followed by serum‐deprivation for 24 h. The efficiency of IFT88 and PTH1R silencing was tested by real time PCR (a). Alternatively, osteocyte cells were serum‐deprived for 24 h and treated with 1 mM aqueous chloral hydrate or with 100 nM PTHrP (7‐34) for 1 h. Cells were subsequently stimulated with shear stress (10 dynes/cm^2^) or with 100 nM PTHrP (1‐37) for 10 min. CM was collected after 18 h (b−d). To evaluate the number of migratory cells, RAW 264.7 (b and c) or human monocytic cells from buffy coat (d) were cultivated in transwell cell culture chamber inserts with an 8 µm pore size. In the lower compartment 20% of each MLO‐Y4 cell‐conditioned medium was added. After 6 h, cells were fixed, stained with crystal violet, and counted with an inverted optical microscope. The number of monocytic cells evaluated with ImageJ software are represented. Results are the mean ± SD of triplicates. *p < 0.05 versus corresponding scrambled siRNA; **p < 0.01 versus corresponding scrambled siRNA; ^a^ *p* < 0.01 versus SC or corresponding IFT88 siRNA/PTH1R siRNA; ^b^ *p* < 0.01 versus SC or corresponding cilium or PTH1R inhibition. CM, conditioned media; FF; fluid flow; IFT88, intraflagellar transport 88 protein; mRNA, messenger RNA; NC, negative control; PTHrP, PTH‐related protein; PTH1R, PTH 1 receptor; SC, static control; SD, standard deviation; siRNA, small interfering RNA.

To assess the role of primary cilia and PTH1R on the effect of mechanically‐stimulated migration, we inhibited the formation of primary cilia in osteocytic cells before FF or PTHrP stimulation using two strategies; silencing IFT88, a protein required for ciliogenesis and primary cilia functional competence (Takei et al., 2018) or preincubation with chloral hydrate, a primary cilia inhibitor (Deren et al., 2016). PTH1R was also inhibited silencing the PTH1R gene, or by using the PTH/PTHrP receptor antagonist PTHrP (7‐34). The efficiency of IFT88/PTH1R silencing was confirmed by RNA assessment in MLO‐Y4 osteocytes (Figure 1a).

After FF or PTHrP stimulation, CM from osteocytes was obtained to be used in migration and osteoclast differentiation assays. Migration of the murine monocyte RAW cell line was drastically inhibited by CM from FF‐ or PTHrP‐stimulated MLO‐Y4 cells compared to migration induced by CM from osteocytes under SC conditions (Figure 1b). In contrast, CM from IFT88‐ or PTH1R‐silenced osteocytes stimulated by either FF or PTHrP showed no inhibitory effects on monocyte migration (Figure 1b). Similarly, the CM of FF‐ or PTHrP‐stimulated osteocytes was unable to decrease murine (Figure 1c) or human (Figure 1d) monocytic cell migration when osteocytes were preincubated with the primary cilium inhibitor chloral hydrate or with the PTH1R inhibitor PTHrP (7‐34).

These data show that the secretome of osteocytes stimulated by FF or PTHrP inhibits the migration of RAW 264.7 and human monocytic cells by a mechanism dependent on the osteocytic primary cilium and PTH1R.

**Figure 2:**
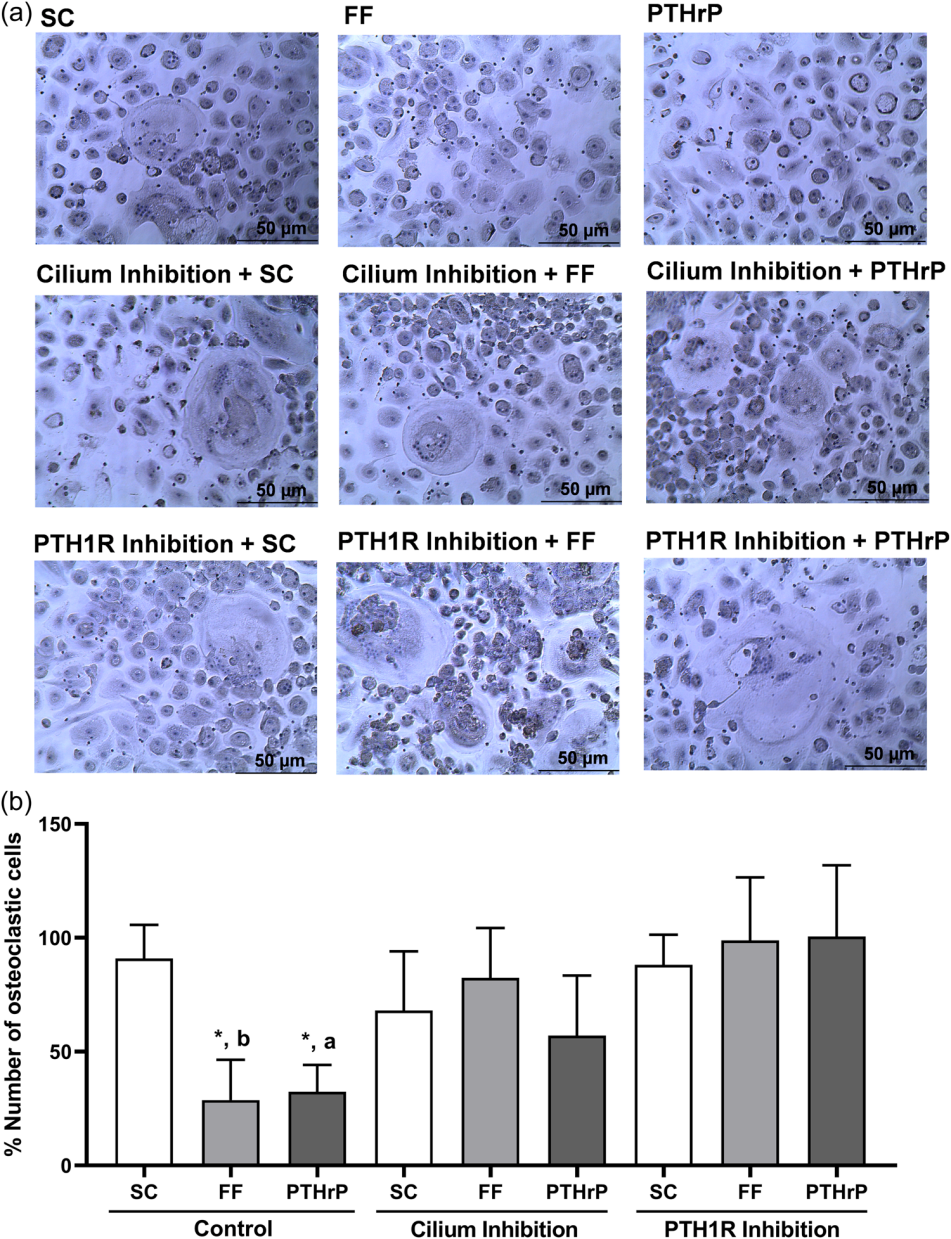
Secretome from mechanically‐stimulated MLO‐Y4 cells reduces the differentiation of human monocytes into osteoclasts by a mechanism dependent on the primary cilium and PTH1R. MLO‐Y4 cells were serum‐deprived for 24 h, treated with 1 mM aqueous chloral hydrate or with 100 nM PTHrP (7‐34) for 1 h and stimulated with shear stress (10 dynes/cm^2^) or with 100 nM PTHrP (1‐37) for 10 min. CM was collected after 18 h. To evaluate the differentiation of monocytic cells into osteoclasts, human monocytes were treated with 20 ng/ml M‐CSF and 20 ng/ml RANKL plus the corresponding 20% MLO‐Y4 cell‐conditioned mediums. Then, cells were fixed, permeabilized, and stained with hematoxylin. The differentiation of human monocytes into osteoclasts was determined by evaluation of the morphology, observing the formation of giant cells (≥50µm) with at least three or more nuclei. Representative images of each condition are shown (a). The percentage of cells with three or more nuclei evaluated with ImageJ software is represented (b). Results are the mean ± SD of triplicates. *p < 0.05 versus SC; **p < 0.01 versus SC; ^a^ *p* < 0.05 versus corresponding PTH1R inhibition; ^b^ *p* < 0.01 versus corresponding cilium or PTH1R inhibition. CM, conditioned media; FF; fluid flow; M‐CSF, macrophage colony‐stimulating factor; PTHrP, PTH‐related protein; PTH1R, PTH 1 receptor; RANKL, receptor activator for nuclear factor κ B ligand; RANKL and (+) α‐MEM medium; SC, static control; SD, standard deviation.

Following, we evaluated the putative actions of osteocytic primary cilia and PTH1R on osteoclast differentiation. We observed that CM obtained from osteocytes stimulated by FF or PTHrP, decreased the percentage of human monocytic cells that differentiate in the presence of osteoclastogenic cytokines into osteoclasts (observed as giant cells with at least three or more nuclei) compared to the percentage of differentiation induced by SC CM (Figure 2a,b). On the other hand, CM of primary cilium‐ or PTH1R‐inhibited MLO‐Y4 osteocytes induced a percentage of osteoclastogenesis that resembled that of CM of osteocytes under SC conditions. These actions were observed even if osteocytes were stimulated with FF or PTHrP (Figure 2a,b). Therefore, these results suggest that the secretome of mechanically‐ and PTHrP‐stimulated osteocytes inhibits the osteoclastogenic process of human monocytes by a mechanism dependent on PTH1R and primary cilium.

### 3.2 PTH1R colocalizes with primary cilium in mechanically‐stimulated osteocytes, being distributed throughout the whole cilium

**Figure 3:**
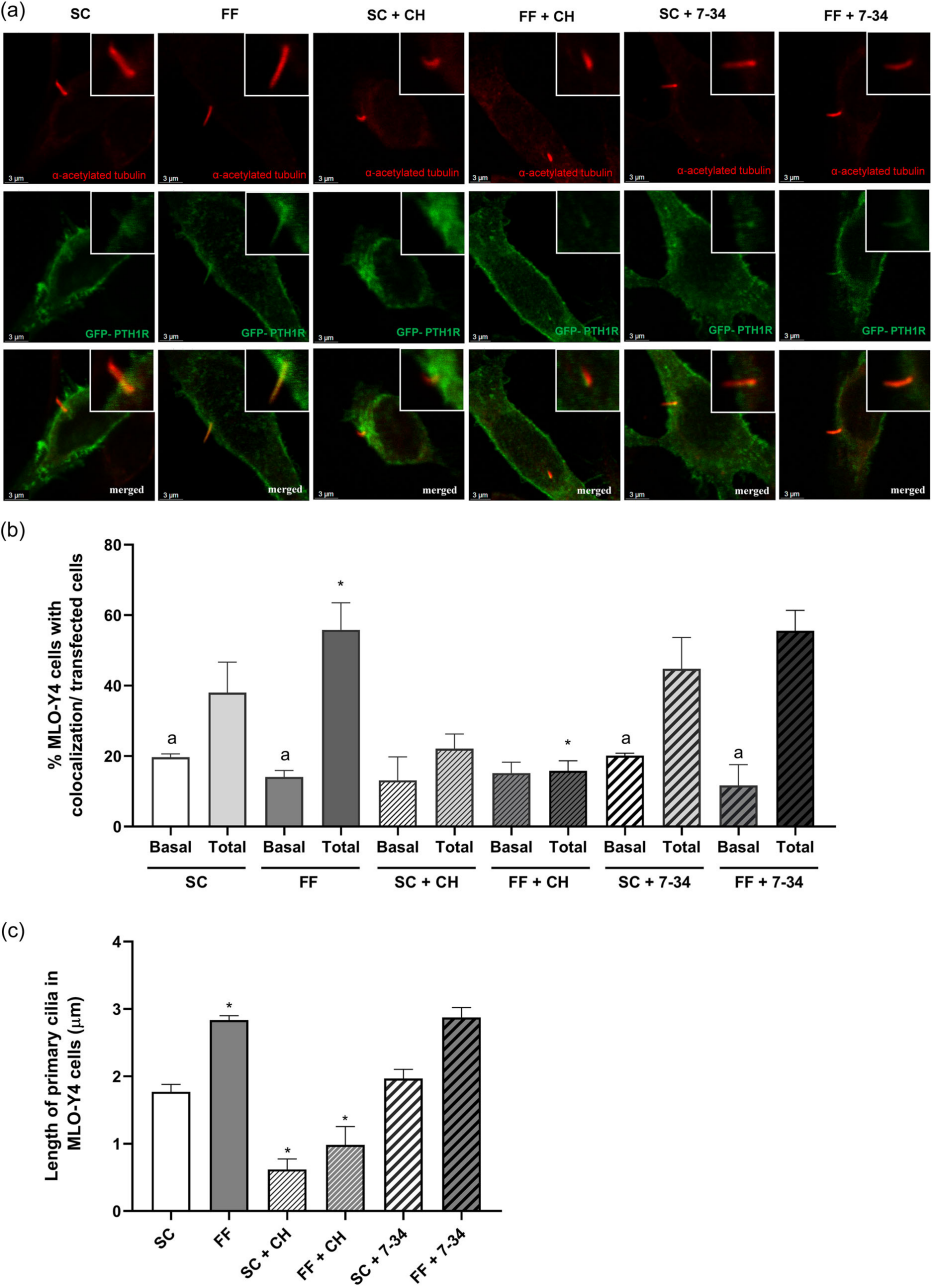
PTH1R colocalizes with primary cilium in mechanically‐stimulated MLO‐Y4 cells, being distributed throughout the whole cilium. MLO‐Y4 cells were transfected with 1 μg of a (GFP)‐PTH1R construct using lipofectamine 3000 for 4 h at 37°C. Cells were subsequently serum‐deprived for 6 h, treated with 1 mM aqueous chloral hydrate or with 100 nM PTHrP (3‐37)  for 1 h and stimulated with shear stress (10 dynes/cm^2^) for 10 min. To evaluate the colocalization of PTH1R with primary cilia, cells were fixed, permeabilized, blocked, and incubated overnight at 4°C with mouse α‐acetylated tubulin antibody. Then, cells were incubated for 1 h with Alexa fluor 546‐conjugated anti‐mouse IgG secondary antibodies. Representative images of each condition are shown (a). The percentage of cells with PTH1R colocalization just at the base (basal) and throughout the whole (total) primary cilium was analyzed in each condition in cells transfected with a GFP‐PTH1R plasmid (b). Evaluation of the length of primary cilia in MLO‐Y4 cells using ImageJ software are represented (c). Results are the mean ± SD of triplicates. *p < 0.05 versus SC or corresponding control; ^a^ *p* < 0.05 versus corresponding total conditions. 7‐34, PTHrP (7‐34); CH, chloral hydrate; FF; fluid flow; (GFP)‐PTH1R, green fluorescent protein‐PTH1R; IG, immunoglobulin G; SC, static control; SD, standard deviation.

We next determined the possible colocalization between the primary cilium and PTH1R in MLO‐Y4 cells and whether their colocalization could impact the functions of both mechanosensors. We found that, in static conditions, 58% of ciliated MLO‐Y4 cells showed certain colocalization of PTH1R and primary cilia. Of those, only 38% of the cells showed colocalization along the entire primary cilium whereas 20% displayed colocalization just at the base of the cilium. However, after FF stimulation, 70% of ciliated osteocytic cells manifested colocalization of PTH1R and primary cilia. Of this percentage, 56% of ciliated cells showed PTH1R localization on the whole extension of the cilium whereas 14% displayed colocalization only at the base of the cilium (Figure 3a,b). CH pretreatment abrogated cilium formation and thus, also PTH1R mobilization to it, while PTHrP (7‐34)  did not affect PTH1R mobilization (Figure 3a,b). Furthermore, the length of primary cilia was increased in mechanically‐stimulated osteocytes compared to shorter length of cilia in osteocytes under SC conditions (Figure 3c). Again, this effect was inhibited by CH pretreatment but was unaffected by the PTH1R antagonist PTHrP (7‐34) (Figure 3c).

Therefore, these results show that mechanical stimulus enlarges osteocyte primary cilia and triggers significant mobilization of PTH1R along the primary cilium of these cells.

### 3.3 Hegdehog, protein kinase A (PKA), and protein kinase C (PKC) pathways mediate osteocyte regulation of monocyte migration and osteoclastogenesis

**Figure 4:**
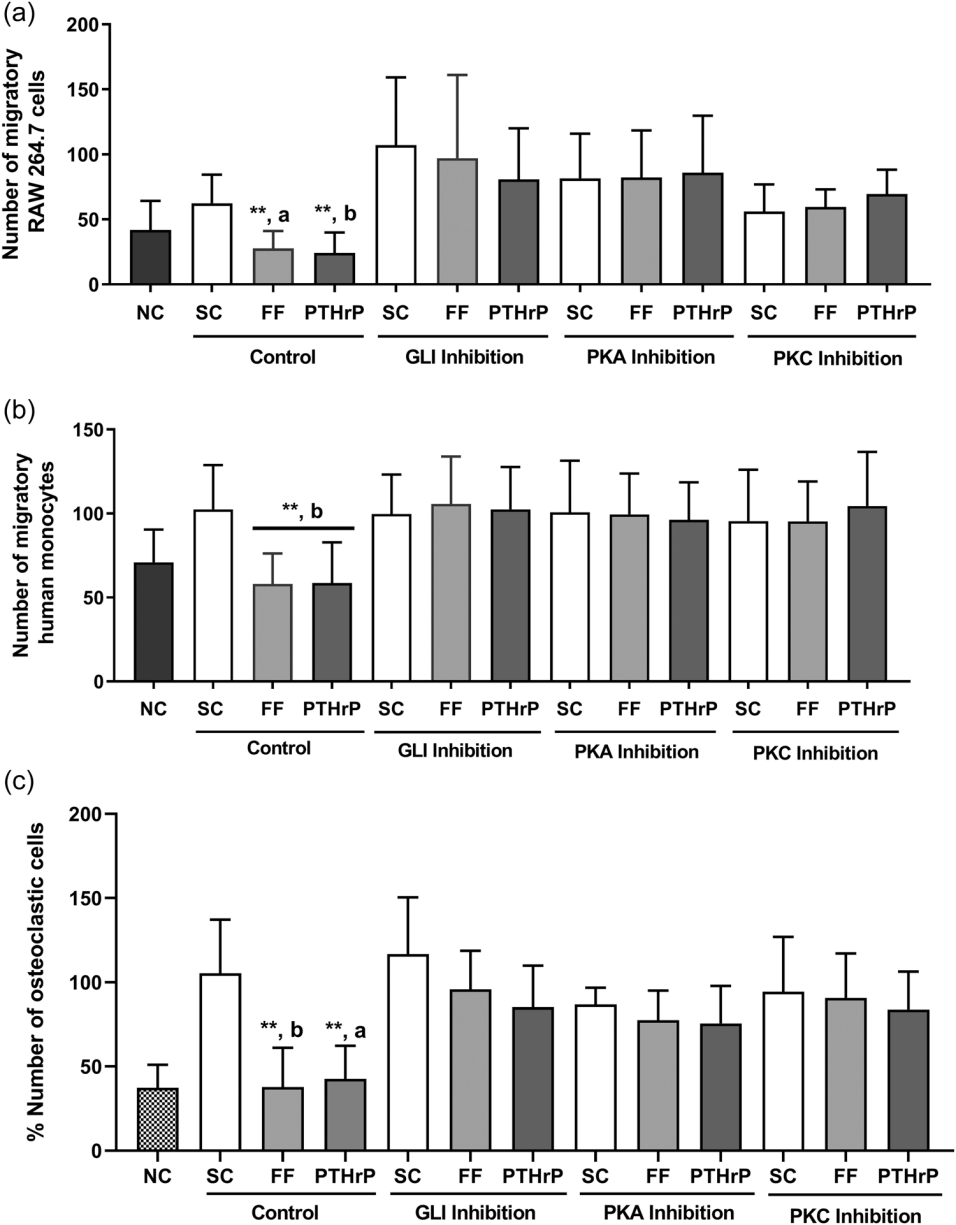
Osteocytic hegdehog, PKA, and PKC signaling pathways are involved in MLO Y4 CM‐dependent regulation of monocyte migration and osteoclastogenesis. MLO‐Y4 cells were serum‐deprived for 24 h and treated with 10 μM GANT61, 100 μM of the adenylate cyclase inhibitor SQ22536, or with 1 μM of the phospholipase C inhibitor U73122 for 1 h. Cells were subsequently stimulated with shear stress (10 dynes/cm^2^) or with 100 nM PTHrP (1‐37) for 10 min. CM was collected after 18 h. To evaluate the number of migratory cells, RAW 264.7 (a) or human monocytic cells from buffy coat (b) were cultured in transwell cell culture chamber inserts with an 8 µm pore size, and in the lower compartment with 20% of each MLO‐Y4 cell‐conditioned medium. After 6 h, cells were fixed, stained with crystal violet, and counted with an inverted optical microscope. The number of monocytic cells evaluated with ImageJ software are represented (a and b). To evaluate the differentiation of monocytes into osteoclasts, human monocytes were treated with 20 ng/ml M‐CSF and 20 ng/ml RANKL, plus the corresponding 20% MLO‐Y4 cell‐conditioned mediums. Cells were fixed, permeabilized, and stained with hematoxylin. The percentage of cells with three or more nuclei evaluated with ImageJ software are represented (c). Results are the mean ± SD of triplicates. **p < 0.01 versus SC; ^a^ *p* < 0.05 versus corresponding GLI, PKA, or PKC inhibition; ^b^ *p* < 0.01 versus corresponding GLI, PKA, or PKC inhibition. CM, conditioned media; FF; fluid flow; GANT61, Gli‐1‐Antagonist 61; M‐CSF, macrophage colony‐stimulating factor; NC, negative control (−) RANKL and (+) α‐MEM medium; PKA, protein kinase A; PKC, protein kinase; PTHrP, PTH‐related protein; RANKL, receptor activator for nuclear factor κ B ligand; SC, static control; SD, standard deviation.

PTH1R actions on bone cells have associated with different signaling pathways including overexpression of Gli transcription factor (Martín‐Guerrero et al., 2020), and PKA and PKC activation (Ardura et al., 2019). To investigate the signaling pathways involved in the regulation of migration and differentiation of osteoclasts by MLO‐Y4 cells CM, we evaluated how the inhibition of PKA, PKC, or hedgehog signaling pathways in osteocytes affects the migration of the murine monocyte line RAW 264.7 cells (Figure 4a) and human monocytic cells (Figure 4b). Pretreatment of osteocytes with Gant61, SQ22536, and U‐73122 (inhibitors of Gli, PKA, and PKC, respectively) decreased the FF‐ and PTHrP‐dependent inhibitory effects of osteocytic CM on murine and human monocyte migration (Figure 4a,b). Next, we studied the effect of the different previously described CMs on osteoclastogenesis. Similarly, CM obtained from FF‐ or PTHrP‐stimulated osteocytes pretreated with the inhibitors Gant61, SQ22536, and U‐73122 completely reversed the repression of osteoclast differentiation caused by noninhibited FF‐ or PTHrP‐stimulated osteocytes (Figure 4c).

Therefore, these data show that stimulation of osteocytes by FF and PTHrP inhibits the migration of monocytic cells and their differentiation toward osteoclasts by an effect dependent on Gli, PKA, and PKC.

### 3.4 Mechanical stimulation inhibits the recruitment and differentiation of osteoclasts through CXCL5 meanwhile PTH1R activation regulates osteoclastogenesis through IL‐6

**Figure 5:**
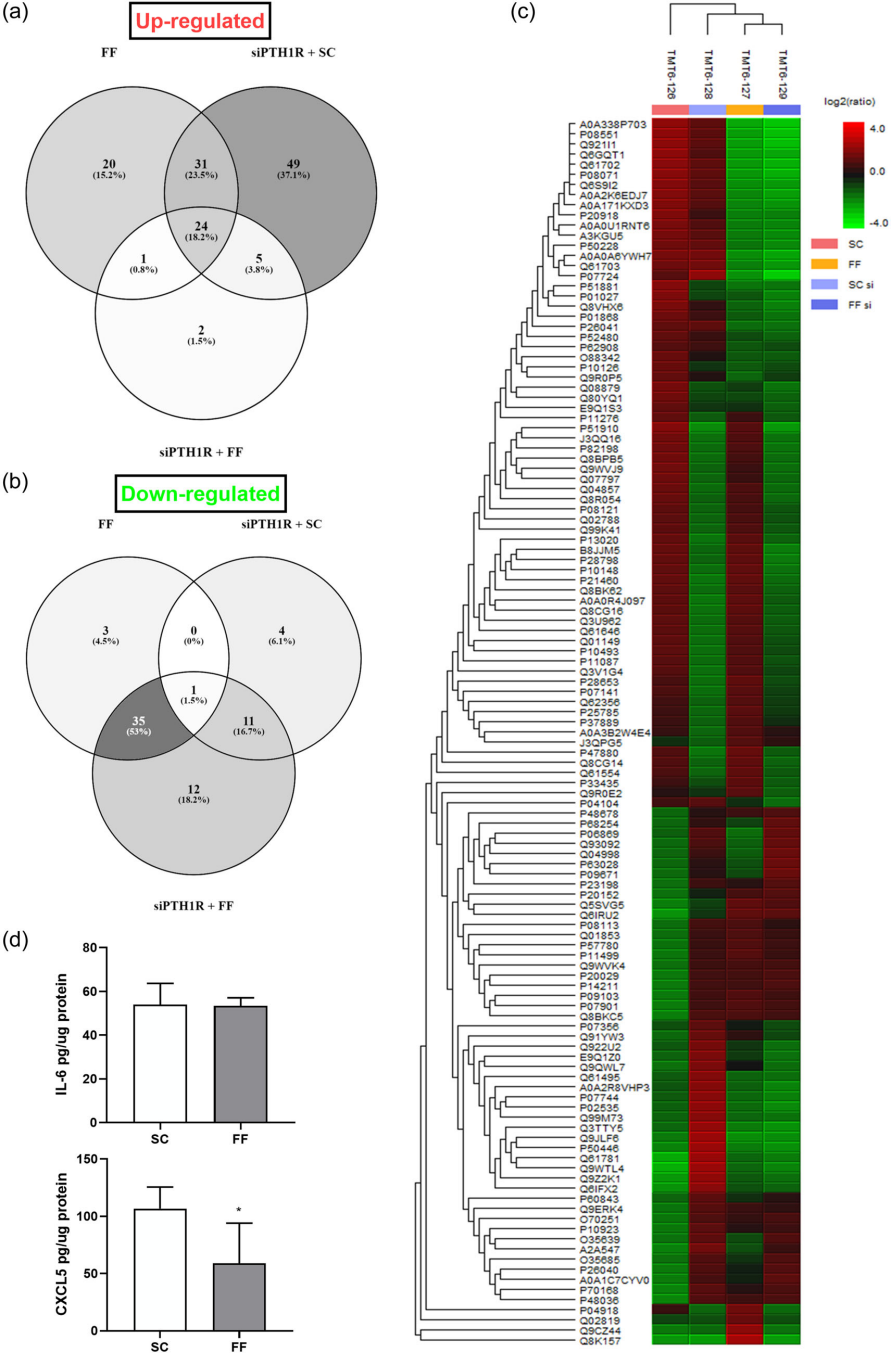
Proteomic analysis of mechanically‐stimulated MLO‐Y4 cells, +/‐ PTH1R silencing. MLO‐Y4 osteocytic cells were transfected with three PTH1R siRNAs or with a scrambled siRNA for 24 h. Cells were subsequently serum‐deprived for 24 h and stimulated with shear stress (10 dynes/cm^2^) for 10 min. CM were collected after 18 h, lyophilized, and analyzed by tandem mass tag mass spectrometry. Venn diagrams show the profile of secreted proteins that increase (a) or decrease (b) comparing different experimental conditions. (c) A heatmap showing protein secretion differences after proteomic analysis of osteocytes is pictured. The color and intensity of the boxes is used to represent changes (not absolute values) of protein secretion. In the picture above, red represents increased secretion and green represents decreased secretion. Black represents unchanged secretion. (d) IL‐6 and CXCL5 in the MLO‐Y4‐CM were analyzed by ELISA as described in Section 2. *p < 0.05 versus SC. CM, conditioned media; FF; fluid flow; IL‐6, interleukin‐6; PTH1R, PTH 1 receptor; SC, static control; siPTH1R, PTH1R silencing.

We next studied the secretome of MLO‐Y4 osteocytes to analyze the role of PTH1R on the secretion of FF‐stimulated or SC osteocytes and unravel proteins secreted by osteocytes that could modulate monocyte migration and osteoclast differentiation. Secretion of 1.323 proteins was revealed in the CM of osteocytes by proteomics analysis. The secretome of FF‐stimulated osteocytes exhibited 76 proteins that increased and 39 proteins that decreased their levels of secretion compared to osteocytes subjected to SC conditions (Figure 5). Venn diagrams show secretome proteomic changes between FF‐stimulated osteocytes, PTH1R silencing + FF and PTH1R silencing + SC conditions. Proteins displaying increased secretion (fold change >1.5) are shown in Figure 5a. A total of 24 proteins are oversecreted in all the studied groups. Moreover, 31 proteins are commonly secreted by FF‐stimulated osteocytes and siPTH1R‐silenced osteocytes in SC conditions; 1 protein (Prelamin‐A/C) by FF‐stimulated osteocytes either with or without PTH1R silencing; and 5 proteins by PTH1R‐silenced osteocytes under FF or SC conditions which are related with the transforming growth factor‐β signaling pathway, plasminogen activating cascade, cholecystokinin receptor signaling pathway, and blood coagulation. Proteins showing decreased secretion (fold change >1.5) are shown in Figure 5b. Only 1 protein, ADP/ATP translocase 2, decreased in all groups. A total of 35 different proteins decreased their secretion in FF‐stimulated osteocytes with versus without PTH1R silencing and 11 proteins in PTH1R‐silenced osteocytes under SC versus FF‐stimulated. Protein secretion variations among the different conditions are also shown in a heat map where the color and intensity represent changes of protein secretion (Figure 5c). Table 1 shows representative proteins obtained from the proteomic analysis, which are related to chemotactic factors and with bone remodeling process. Some of the proteins overexpressed by osteocytes of this study, were related to inflammatory processes or to recruitment of osteoclast precursors, such as CXCL5 and IL‐6. In this regard, the proteomics data showed that FF inhibits the secretion of CXCL5, but not IL‐6. In contrast, the silencing of PTH1R induced IL‐6 secretion without affecting CXCL5 secretion. To validate these results, we determined IL‐6 and CXCL5 levels in MLO‐Y4 CM after SC and FF conditions. We confirmed that FF did not affect IL‐6 secretion but decreased CXCL5 levels (Figure 5d).

**Table 1:** Protein secretion differences after proteomic analysis of mechanically‐stimulated MLO‐Y4 cells, +/‐ PTH1R silencing are pictured

	Gene	Protein	Significance (%)	Ratio SC	Ratio FF	Ratio siPTH1R SC	Ratio siPTH1R FF
Chemokines factors	*CCL2*	C‐C motif chemokine 2	19.57	1	0.99	0.51	**0.48**
	*CXCL3*	C‐X‐C motif chemokine 3	8.39	1	1.17	1.44	1.27
	*CXCL5*	C‐X‐C motif chemokine 5	32.82	1	**0.41**	0.9	**0.33**
	*CCL7*	C‐C motif chemokine 7	8.32	1	1.15	0.79	0.63
	*C3*	Complement C3	41.02	1	**0.46**	0.51	**0.32**
	*IL‐6*	Interleukin‐6	6.86	1	1.11	**1.64**	**1.94**
Pro‐plasmin factors	*A2M*	Alpha‐2‐macroglobulin‐P	90.18	1	**0.2**	0.58	**0.16**
	*PLG*	Plasminogen	14.9	1	**0.32**	0.6	**0.31**
	*PLAU*	Urokinase‐type plasminogen activator	19.33	1	0.89	**1.59**	**1.79**
	*Serpinc1*	Antithrombin‐III	53.91	1	**0.29**	0.95	**0.21**
Pro‐anabolic factors	*AHSG*	Alpha‐2‐HS‐glycoprotein	63.08	1	**0.16**	0.62	**0.1**
	*DKK3*	Dickkopf‐related protein 3	11.45	1	**1.52**	0.66	0.84
Calcium‐binding proteins	*ANXA1*	Annexin A1	12.51	1	1.49	**1.66**	1.45
	*ANXA2*	Annexin A2	13.02	1	1.19	**1.56**	1.03
	*ANXA3*	Annexin A3	21.3	1	1.33	1.99	1.84
	*ANXA5*	Annexin A5	27.28	1	**2.09**	**2.29**	**2.23**
	*CALM1*	Calmodulin‐1	10.14	1	1.38	1.71	**1.47**
	*CALM2*	Calmodulin‐2	10.14	1	1.38	1.71	**1.47**
	*CALM3*	Calmodulin‐3	10.14	1	1.38	1.71	**1.47**
	*NUCB1*	Nucleobindin‐1	16.91	1	**1.58**	0.95	0.95
Transmembrane signal receptors	*INSRR*	Insulin receptor‐related protein	42.17	1	**2.36**	**8.06**	**1.77**
	*THBD*	Thrombomodulin	4.6	1	1.42	1.33	**1.67**

*Note*: The table shows the changes for each protein and the ratio for each condition relativized versus static control. Increased and decreased secretion values are shown in bold.

Abbreviations: CM, conditioned media; FF; fluid flow; PTH1R, PTH 1 receptor; SC, static control; siPTH1R, PTH1R silencing.

**Figure 6:**
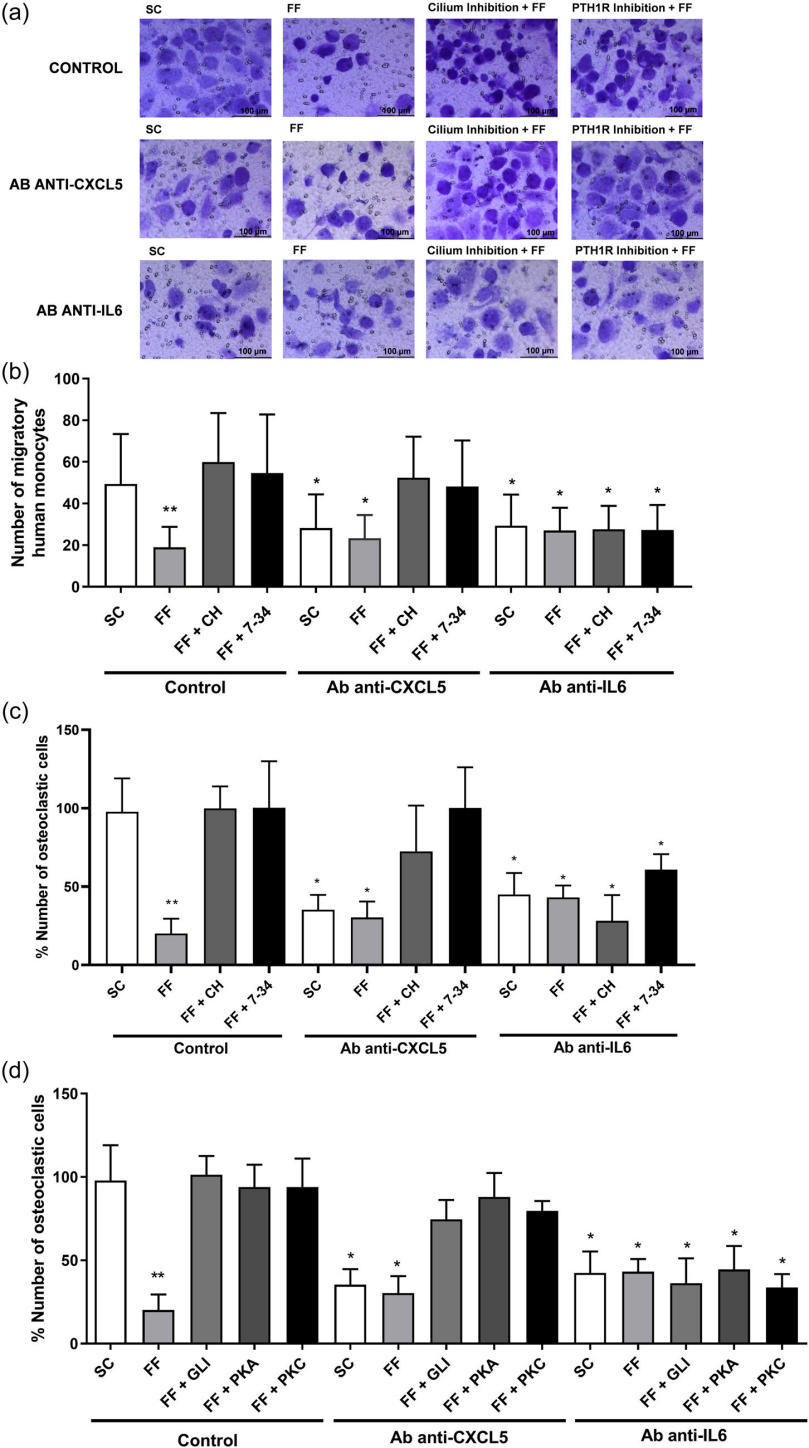
Mechanical stimulation inhibits the recruitment and differentiation of osteoclasts through a mechanism dependent on CXCL5 and IL‐6. MLO‐Y4 cells were serum‐deprived for 24 h and treated with 1 mM aqueous chloral hydrate, 100 nM PTHrP (7‐34), 10μM of the Gli inhibitor GANT61, 100 μM of the adenylate cyclase inhibitor SQ22536 or with 1 μM of the phospholipase C inhibitor U73122, for 1 h. Cells were subsequently stimulated with shear stress (10 dynes/cm^2^) for 10 min. CM were collected after 18 h and 2 µg/ml of neutralizing antibodies anti‐mCXCL5 or 1 µg/ml of anti‐mIL‐6 were added. To evaluate the number of migratory cells, human monocytes from buffy coat (a and b) were cultured in transwell cell culture chamber inserts with an 8 µm pore size. In the lower compartment 20% of each MLO‐Y4 cell‐conditioned medium was added plus the corresponding neutralizing antibody. After 6 h, cells were fixed, stained with crystal violet, and counted with an inverted optical microscope. Representative images of each condition are shown (a). The number of monocytic cells evaluated with ImageJ software are represented (b). To evaluate the differentiation of monocytes into osteoclasts, human monocytes were treated with 20 ng/ml M‐CSF and 20 ng/ml RANKL, plus the corresponding treatments: 20% of each MLO‐Y4 cell‐conditioned medium and the neutralizing antibody. Cells were fixed, permeabilized, and stained with hematoxylin (c and d). The percentage of cells with three or more nuclei evaluated with ImageJ software is represented (c and d). Results are the mean ± SD of triplicates. *p < 0.05 versus SC; **p < 0.01 versus SC. 7‐34, PTHrP (7‐34); Ab, antibody; CH, chloral hydrate; CM, conditioned media; CXCL5, C‐X‐C motif chemokine 5; FF; fluid flow; GANT61, Gli‐1‐Antagonist 61; IL‐6, interleukin‐6; M‐CSF, macrophage colony‐stimulating factor; PKA, protein kinase A; PKC, protein kinase C; RANKL, receptor activator for nuclear factor κ B ligand; SC, static control; SD, standard deviation.

Based on these observations, we analyzed whether IL‐6 and CXCL5 were involved in the regulation of osteoclast migration and differentiation induced by PTH1R‐ and primary cilia‐dependent mechanisms in MLO‐Y4 cells using specific neutralizing antibodies (Figure 6). As previously shown, CM from osteocytes under SC caused an increase of monocyte migration and osteoclast differentiation; an effect that was inhibited by either pretreatment of CM of osteocytes with an anti‐CXCL5 or an anti‐IL‐6 antibody. In addition, FF decreased monocyte migration and osteoclast differentiation with or without CXCL5 or IL‐6 neutralization (Figure 6). However, CXCL5 neutralization caused no effect on monocyte migration or osteoclast differentiation when primary cilium or PTH1R from osteocytes were inhibited by CH or PTHrP (7‐34), respectively (Figure 6a−c). Conversely, anti‐IL‐6 not only reversed migration (Figure 6a,b) and osteoclastogenesis (Figure 6c) in SC conditions, but also in the presence of CH or PTHrP (7‐34) (Figure 6a−c). In this regard, osteoclastogenesis processes induced by the inhibition of Gli, PKA, or PKC pathways in FF condition were reversed by IL‐6 neutralization but not by the preincubation with CXCL5 antibody (Figure 6d).

**Figure 7:**
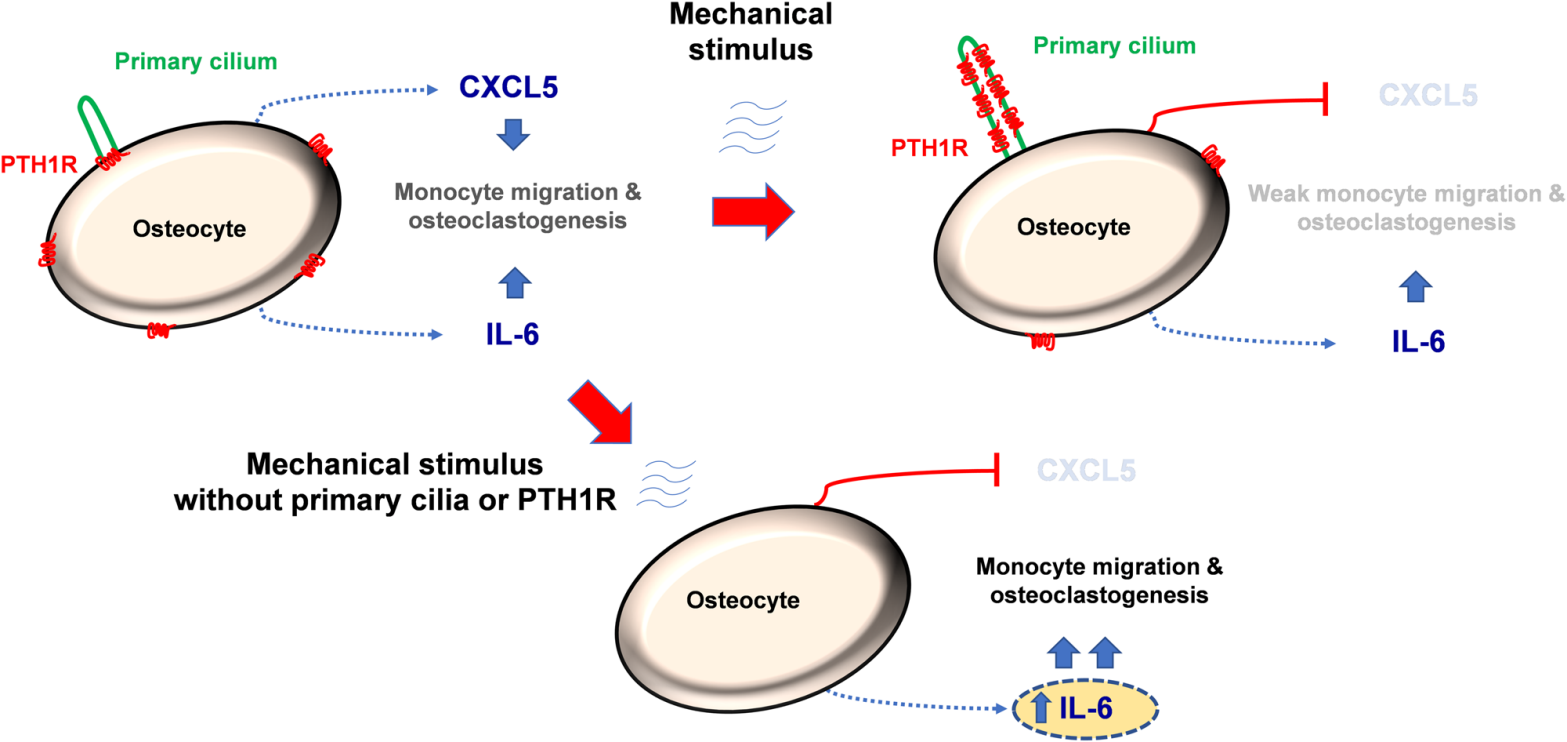
Proposed mechanism for regulation of osteoclast recruitment and differentiation by primary cilium and PTH1R in osteocytes. The presence of both functional primary cilium and PTH1R in osteocytes are necessary for correct communication with osteoclasts. Mechanical stimulation inhibits the recruitment and differentiation of osteoclasts through CXCL5, while PTH1R activation regulate osteoclasts through IL‐6. CXCL5, C‐X‐C Motif Chemokine Ligand 5; IL‐6, interleukin‐6; PTH1R, parathyroid hormone receptor type 1.

These findings indicate that the presence of both functional primary cilium and PTH1R in osteocytes are necessary for correct communication with osteoclasts and suggest that mechanical stimulation inhibits the recruitment and differentiation of osteoclasts through CXCL5, while PTH1R activation regulate osteoclasts through IL‐6 (Figure 7).

## 4 DISCUSSION

Osteocyte‐osteoclast communication is a key process for the physiological maintenance of bone homeostasis. In the present study, we show that PTH1R and primary cilia are part of the molecular machinery responsible for transducing mechanical stimulation in order to decrease osteoclastic cell migration and differentiation, via regulation of CXCL5 and IL‐6 secretion.

PTH1R is expressed in osteoblast lineage cells in bone and is needed for normal bone formation and remodeling (Delgado‐Calle, Xioalin et al., 2017). PTH1R signaling exerts direct effects on osteocytes to promote bone formation via suppression of sclerostin and through the induction of RANKL to increase osteoclastic resorption (Estell & Rosen, 2021). The two natural agonists of PTH1R are PTH, secreted by parathyroid glands, and PTHrP, which is secreted by a wide variety of tissues where it acts locally (Ardura et al., 2019). PTH1R activation can induce bone anabolism, after an intermittent administration of PTH or PTHrP, or catabolism, after sustained PTH elevation (Ardura et al., 2019; Maycas et al., 2017; Rhee et al., 2011; Tu et al., 2015). In the last few years, several studies have shown that PTH1R might be activated directly by mechanical stimulation. Zhang & Frangos, (2009) demonstrated that shear stress leads to ligand‐independent conformational changes of the PTH1R in osteoblastic MC3T3‐E1 cells. Interestingly, PTH and mechanical stimulation exert different conformational changes of the receptor, suggesting that although both stimuli may stimulate PTH1R, the mechanisms underlying activation of the receptor and its intracellular responses may also be different (Zhang & Frangos, 2009). In this regard, a previous study from our group showed that mechanical stimulation by hypotonic shock or FF activates PTH1R in a ligand independent manner (Maycas et al., 2015b). In that study, we described that mechanical stretching rapidly (<1 min) stimulated Ca_i_^2+^ transients in PTH1R‐overexpressing HEK‐293 cells and in MLO‐Y4 cells. Moreover, calcium signaling was unaffected after PTHrP gene silencing but was inhibited by knocking down PTH1R (Maycas et al., 2015b). On the other hand, it has also been described that mechanical loading such as physical exercise and PTH/PTHrP peptides can synergically potentiate each other actions (Gardinier et al., 2016, 2019; Maycas et al., 2017; Robinson et al., 2021; Sugiyama et al., 2008; Y. Wang et al., 2007). In fact, physical activity as running or swimming induces transient PTH secretion (Gardinier et al., 2016), and mechanical stimulation increases PTHrP production in osteocytes, which might induce PTH1R activation (Chen et al., 2005; Maycas et al., 2015a). In a similar way, the anabolic action of PTH or PTHrP is enhanced by mechanical loading (Maycas et al., 2017; Sugiyama et al., 2008).

Primary cilia act as mechanosensors, receiving mechanical signals and transducing them through their specific machinery. The primary cilium organelle is a nonmotile unique protrusion of the cell membrane that is formed during the G0 phase of the cell cycle and functions as an antenna. There are a great number of receptors and ion channels that bind to the membrane of the primary cilium. Moreover, primary cilium has been shown to mediate mechanical loading‐induced bone formation (Martín‐Guerrero et al., 2020; Rais et al., 2015) by coordinating bone‐forming osteoblast proliferation and continuous replenishment of osteoblasts from mesenchymal stem cell populations in response to paracrine factors secreted by mechanosensitive osteocytes (Hoey et al., 2011; Leucht et al., 2013). This organelle has also been involved in mechanically‐stimulated recruitment of osteoblasts and posterior differentiation into embedded, fully mature osteocytes (Hoey et al., 2011; Moore et al., 2019).

We have recently shown that PTH1R localizes into primary cilia, both in control or PTHrP‐stimulated conditions in osteocyte and osteoblast cells (Martín‐Guerrero et al., 2020). Moreover, a recent study demonstrated that mechanical stress promotes transport of PTH1R to primary cilia, increasing PTH signaling in nucleus pulposus cells from the intervertebral disc (Zheng et al, 2018). However, to our knowledge, PTH1R redistribution along the whole primary cilium after mechanical stimulation by FF in bone cells has not been described before. The present data suggest that mechanical stimulation may enhance PTHrP effectiveness by potentiating a more accessible distribution of its receptor, PTH1R, to external mechanical stimuli such as PTH1R natural agonists. These could explain, at least in part, the therapeutic efficiency of combined PTH or PTHrP treatment and mechanical stimulation to preserve bone mass and quality.

Mechanical loading plays an essential role in the maintenance of bone quality and quantity, and osteocytes are critical for sensing mechanical stimulation (Bellido, 2014; Choy et al., 2020). Osteocytes respond to mechanical stimulation by releasing proteins that regulate osteoclast and osteoblast activity, including Sclerostin, RANKL, osteoprotegerin (OPG), and M‐CSF. These paracrine factors can regulate osteoclast differentiation from monocyte precursors (Delgado‐Calle, Sato et al., 2017; Wijenayaka et al., 2011; You et al., 2008). In this regard, RANKL and M‐CSF promote osteoclast differentiation and activity (Takayanagi, 2007). Osteocyte‐like MLO‐Y4 cells promote osteoclast formation by increasing surface bound RANKL and secreting M‐CSF (Simfia et al., 2020; Xiong et al., 2011; Zhao et al., 2002). WNT/β‐catenin signaling pathway regulates production of RANKL, M‐CSF, and OPG by osteocytes. Differentiated osteoblasts also regulate osteoclast formation by activating β‐catenin/Wnt pathway, that inhibits the secretion of the RANKL decoy molecule, OPG (Baron & Kneissel, 2013). Other osteocyte‐derived factors that contribute to osteoclast differentiation and function include IL‐6, tumor necrosis factor alpha (perhaps through osteocyte‐derived apoptotic bodies), and high mobility group box 1 (Robling & Bonewald, 2020). Many inflammatory cytokines regulate osteoclastogenesis. For example, IL‐4 and IL‐10 inhibit osteoclast formation whereas stromal cell‐derived factor‐1 and monocyte chemoattractant protein‐1 stimulate it (H. R. Kim et al., 2014; M. S. Kim et al., 2006; Takayanagi, 2009). While the field of osteocyte control of osteoclastogenesis has made great strides in recent years, several RANKL/OPG independent mechanisms are currently under investigation (O'brien et al., 2016). In the present study, we have found that mechanical stimulation by FF inhibits preosteoclast migration and differentiation by a mechanism dependent of PTH1R and primary cilia.

Proteomic analysis and neutralizing experiments point to CXCL5 as a cytokine regulated by osteocytes that, when is secreted, controls osteoclast migration and differentiation. CXCL5 is a chemokine involved in leukocyte recruitment (Nouailles et al., 2014; Yoshida et al., 2014). This chemokine binds to CXC receptor 1 and CXC receptor 2, which are both expressed in osteoclast precursors (Grassi et al., 2003; Li et al., 2011). Moreover, CXCL5 has been describe to increase RANKL expression in Paget's disease in bone (Sundaram et al., 2013). Other study shows that microgravity promotes osteoclast formation through the induction of syncytin‐A expression in RAW264.7 preosteoclast cells without RANKL stimulation. The effects of osteotropic factors such as CXCL5 to regulate the expression of syncytin‐A in preosteoclast cells subjected to microgravity compared to ground based cultures has been confirmed (Ethiraj et al., 2018). However, in the present study when PTH1R gene expression was silenced CXCL5 was not affected, although FF effects on osteoclast migration and differentiation were completely reversed. This observation suggests that secretion of CXCL5 is highly dependent on mechanical stimulation but does not change whether PTH1R is activated or not.

We also observed that IL‐6 secretion was increased when PTH1R was silenced, both in static and FF conditions. Previous studies found that IL‐6 enhances osteocyte‐mediated osteoclastogenesis by promoting JAK2 and RANKL activity (Wu et al., 2017). Kazuhiro's study demonstrated that a combination of TNF and IL‐6 can induce bone‐resorptive activity in osteoclast‐like cells (Yokota et al., 2014). Indeed, IL‐6 plays a significant role as a biochemical regulator during osteoclastogenesis and bone resorption and regeneration (Cheung et al., 2012). Similar to this study, osteoclast formation was enhanced after the secretion of both IL‐6 and soluble IL‐6 receptor (Palmqvist et al., 2002). Accordingly, we show herein that IL‐6 neutralizing antibody decreased cell migration and differentiation when PTH1R was inhibited. However, CXCL5 neutralizing antibody had no effect on this regard.

We have observed that PTH1R silencing is associated with increased monocyte migration and osteoclastogenesis and also with IL‐6 over‐secretion by osteocytes even when cells were stimulated by FF. Given that IL‐6 neutralization under these conditions decreases both migration and osteoclasts differentiation, these data suggest that secretion of high levels of IL‐6 can overcome FF‐dependent under‐secretion of CXCL5 and maintain monocyte migration and osteoclastogenesis. In a similar way, we showed that primary cilia inhibition is also associated with increased osteoclast function, even in mechanical stimulated conditions, and IL‐6 neutralization reversed this effect. Collectively, these data suggest that abrogation of primary cilia might induce an IL‐6 over‐secretion that overcome CXCL5 under‐secretion, as occur when PTH1R is silenced. However, it is also possible that primary cilia under FF condition could modulate other alternative cytokines involved in osteoclast communication. Still, IL‐6 neutralization was enough to avoid migration and differentiation of osteoclast precursors.

Our findings support that functional primary cilium and PTH1R are required in osteocytes to regulate the secretome of these cells and their communication with osteoclasts. Thus, mechanically stimulated‐osteocytes inhibit osteoclast recruitment and differentiation by decreasing CXCL5 secretion, while activation of osteocytic PTH1R regulate osteoclasts via modulation of Il‐6 secretion.
